# Vitamin D and respiratory tract infections in childhood

**DOI:** 10.1186/s12879-015-1196-1

**Published:** 2015-10-28

**Authors:** Susanna Esposito, Mara Lelii

**Affiliations:** Pediatric Highly Intensive Care Unit, Department of Pathophysiology and Transplantation, Università degli Studi di Milano, Fondazione IRCCS Ca’ Granda Ospedale Maggiore Policlinico, Via Commenda 9, 20122 Milan, Italy

**Keywords:** Acute otitis media, Bronchiolitis, Community-acquired pneumonia, Pharyngotonsillitis, Respiratory tract infection, Rhinosinusitis, Vitamin D, Vitamin D supplementation

## Abstract

**Background:**

Respiratory tract infections (RTIs) remain among of the most important causes of morbidity and mortality among children. Several studies have associated vitamin D deficiency with an increased risk of RTIs, and vitamin D supplementation has been proposed as a possible preventive measure against RTIs in children. The main aim of this review is to summarize the current evidence from the literature about the link between vitamin D and RTIs in children.

**Discussion:**

Several recent studies have shown that vitamin D has different immunomodulatory properties associated with the risk of RTIs in childhood. In this regard, it is very important to understand the definition of deficiency and insufficiency of vitamin D and when and how to treat this condition. Unfortunately, there is no consensus, although a level of at least 10 ng/mL 25-hydroxycholecalciferol (25[OH]D) is thought to be necessary to promote bone mineralization and calcium homeostasis, and a concentration between 20 ng/mL and 50 ng/mL is considered adequate to provide an immunomodulatory effect. Available data support a role for vitamin D deficiency in the risk of pediatric tuberculosis, recurrent acute otitis media, and severe bronchiolitis, whereas further studies are needed to confirm an association in children with recurrent pharyngotonsillitis, acute rhinosinusitis and community-acquired pneumonia.

**Conclusions:**

Maintenance of adequate vitamin D status may be an effective and inexpensive prophylactic method against some RTIs, but the supplementation regimen has not been clearly defined. Further clinical trials are needed to determine the 25(OH)D concentrations associated with an increased risk of RTIs and optimal vitamin D supplementation regimen according to the type of RTI while also taking into consideration vitamin D receptor polymorphisms.

## Background

Vitamin D, or the “sunshine vitamin,” is not just a vitamin; it is also a prohormone with numerous functions in the body [1]. “Prohormone” refers to a group of fat-soluble secosteroids. The two major forms are vitamin D2, or ergocalciferol, and vitamin D3, or cholecalciferol [2]. The best-understood function of vitamin D is in the absorption of calcium from the small intestine, which helps to prevent diseases such as osteoporosis and osteomalacia in adults and rickets in children [3–7]. In addition to its important role in skeletal development and maintenance, there is increasing evidence that vitamin D has a beneficial effect on extraskeletal tissues. Tissues such as the brain, heart, stomach, pancreas, lymphatics, skin, gonads, and prostrate tissue are composed of cells, including T and B lymphocytes, that express the vitamin D receptor (VDR). In these tissues, vitamin D is thought to have roles in the improvement of immune function and the reduction of inflammation [8, 9]. Accordingly, there is accumulating evidence that consumption of vitamin D may reduce respiratory tract infection (RTI) susceptibility in children [10, 11]. Initially, the prototypical disease link was tuberculosis (TB), but there are now studies that support a connection with several others RTIs, such as acute otitis media (AOM), pharyngotonsillitis, rhinosinusitis, bronchiolitis and pneumonia [12, 13]. The aim of this review is to describe the evidence in the literature of the link between vitamin D and RTIs in children.

## Discussion

### Vitamin D metabolism

Vitamin D2 (ergocalciferol) and vitamin D3 (cholecalciferol) can be ingested from different types of food (Table 1), or they can be synthesized through exposure to ultraviolet radiation B (UVB) [14]. Skin synthesis usually contributes 80 to 90 % of an individual’s vitamin D, but it depends on several factors. For example, people with darker skin have higher levels of the pigment melanin, which reduces the skin’s ability to produce vitamin D after sun exposure [15]. People at risk for vitamin D deficiency include those with limited sun exposure and those with fat malabsorption [16]. Subjects living in the northern latitudes, homebound individuals, and women who wear long robes and head coverings for religious reasons may also not obtain adequate levels of vitamin D from sunlight [16]. Finally, after the age of fifty, the skin loses its ability to efficiently synthesize vitamin D and the kidneys also convert less to its active form [17–20]. Both the ingested and UVB-synthesized forms of vitamin D are biologically inactive; activation requires hydroxylation in the liver and kidney. In the liver, cholecalciferol is converted to calcidiol (also known as 25-hydroxycholecalciferol, 25[OH]D), whereas ergocalciferol is converted to 25-hydroxyergocalciferol [21]. Part of the calcidiol is converted by the kidneys to calcitriol (1,25-dihydroxyvitamin D3, 1,25[OH]D), the biologically active form of vitamin D; the conversion is controlled by the parathyroid glands [22]. Calcitriol circulates as a hormone in the blood, regulating several mechanisms, and its level depends on the number of nephrons, high serum concentrations of fibroblast growth factor-23, and the level of inflammatory cytokines [23]. Calcitriol is also produced in tissues such as vascular smooth muscle cells, bowel cells, monocytes, dendritic cells (DCs), and B lymphocytes [24–30].

**Table 1:** The principal foods containing vitamin D

Main food sources of vitamin D	
Fatty fish (e.g., tuna, salmon, mackerel)	
Liver	
Cheese	
Milk (fortified)	
Egg	
Other fortified foods (e.g., cereals)	

### VDRs and polymorphisms

The effects of vitamin D are mediated by a cytosolic receptor called VDR. VDR is nearly ubiquitously expressed, and this ubiquity accounts for the numerous and varied mechanisms that are regulated by vitamin D [31]. After its production, 1,25(OH)D binds VDR and then enters the cell nucleus, acting as a ligand-activated transcription factor and activating gene expression [32, 33]. The VDR gene, which is located on chromosome 12q13.1, has several polymorphic regions, some of which are associated with a predisposition for certain diseases [34]. This means that not only is vitamin D deficiency is associated with a considerable risk of diseases, but there is wide interindividual variation in vitamin D sensitivity, which may influence disease risk. Until now, only a few polymorphisms have been studied, and a complete understanding of these complex mechanisms has been lacking [35]. This means that future studies should focus on the functional effects of genetic variations in VDRs and their association with RTIs among pediatric patients with different characteristics.

### Vitamin D as an immune system regulator

Vitamin D has an important influence on the host’s immune system, modulating both innate and adaptive immunity and regulating the inflammatory cascade [36–38]. The hypothesis of the immunoregulatory role of vitamin D derives from the discovery that there are several interactions between vitamin D and the immune system. The majority of immune cells express VDRs, mainly after they themselves have been stimulated [39]. The mechanism by which vitamin D regulates inflammation and immunity appears to be pleiotropic; it controls macrophage and dendritic cell activities and various Toll-like receptor-mediated events in neutrophils [39], and it diminishes the function of human dendritic cells by decreasing maturation, antigen presentation and the production of cytokines such as interleukin (IL)-12 and IL-23 [40]. Moreover, treating macrophages with 1,25(OH)D results in the expression of various cytokines and chemokines, including CXCL8, IL-6, and IL-12, and tumor necrosis factor (TNF)-α [41, 42]. Additionally, vitamin D induces the expression of two antimicrobial peptides—cathelicidin and β-defensin—that are widely expressed in the body and play a key role in innate immunity owing to their chemotactic action and toxin neutralization [37, 38]. Vitamin D shifts cytokine expression from a type-1 to a type-2 phenotype: it represses the transcription of genes encoding type-1 cytokines (connected with Th1-driven autoimmune responses) and Th17-associated cytokines (linked to tissue damage and inflammation) in order to polarize CD4+ T-cells responses toward more regulatory type-2 or Treg phenotypes [43–45]. Thus, 1,25(OH)D seems to contribute to the maintenance of self-tolerance by enhancing protective innate responses. Finally, more evidence of a connection between inflammation and vitamin D comes from a recent study that demonstrated that VDR polymorphisms play a role in obesity that is associated with degrees of ongoing inflammation, possibly resulting from alterations in gut permeability and microbial translocation [46].

With this background, several studies have evaluated whether vitamin D deficiency is associated with an increased risk of RTIs in children [12, 13, 47, 48].

### Vitamin D and respiratory infections in children

In children, infections are a major cause of morbidity and mortality [49, 50]. Numerous studies have identified an association between inadequate vitamin D concentrations and RTIs in children [51]. Initially, an association between vitamin D deficiency and RTIs in children was found after a higher incidence of respiratory infections was found among infants and children with rickets [51]. The increased incidence of RTIs in these children was probably caused by both compromised lung compliance due to the rib deformities associated with severe rickets and poor nutritional status.

Later, the prototypical example of a connection between vitamin D insufficiency and susceptibility to infectious disease was found to be TB; studies published over the past twenty years have noted the link between decreased serum calcitriol concentrations and increased severity and/or susceptibility to TB infection [52, 53].

Gradually, other RTIs in children have also been linked to vitamin D. The evidence that the peak of viral infections is in the winter months when synthesis of vitamin D across the skin is naturally impaired supported the association [54]. In addition, vitamin D deficiency in pregnant women may result in an increased risk of RTIs in their infants. It has been shown that serum 25(OH)D levels during pregnancy can condition the expression of certain tolerogenic genes connected with diseases other than congenital rickets [55]. Thus, vitamin D supplementation during pregnancy appears to have a beneficial effect on children’s health.

#### Vitamin D and TB

In 2000, a case–control study of the Gujarati Indian population in London found that serum vitamin D deficiency was more common in patients with active TB (67 %) than in their uninfected co-inhabitants (26 %; odds ratio [OR] 0.68, with a 95 % confidence interval [CI] 0.43-0.93) who served as the control group [53]. The seasonality of TB observed in several countries in Europe and South Africa [56–58] was considered to be proof of this link. The mechanisms through which vitamin D modulates the immune system in response to *Mycobacterium tuberculosis* infection are not completely understood. Some studies have demonstrated that calcitriol induces anti-mycobacterial activity in vitro by modulating the host response to mycobacterial infection [59–64]. These studies have shown that vitamin D induces reactive nitrogen and oxygen, which inactive matrix metalloproteinase enzymes (MMPs) implicated in the pathogenesis of pulmonary cavitation, with calcitriol decreasing tissue damage by inhibiting MMPs [59–64]. Vitamin D also induces the antimicrobial peptide cathelicidin, which stimulates autophagy of *M. tuberculosis* [59–64]. However, there are interindividual differences that are determined by VDR polymorphisms. The *TaqI* VDR polymorphism has been associated with an increased phagocytosis of *M. tuberculosis* by vitamin D in vitro and a more rapid sputum culture conversion in patients with pulmonary TB [65, 66]. By contrast, the *FokI* VDR polymorphism reduced this antimicrobial activity [66, 67].

More than 20 years ago, Davies et al. proposed that vitamin D would be more effective as a treatment for latent TB infection rather than for active disease [67, 68]. However, a recent multicenter randomized controlled trial in which 146 patients were allocated to receive 2.5 mg vitamin D or placebo at baseline and 14, 28, and 42 days after starting standard TB treatment demonstrated that vitamin D did not significantly affect time to sputum culture conversion in the entire study population but significantly hastened sputum culture conversion in participants with the *TT* genotype of the *TaqI* VDR polymorphism [69]. Moreover, low serum levels of 1,25(OH)D were associated with a higher risk of developing multidrug-resistant TB (MDR TB) [70]. Some recent clinical observational studies have shown that vitamin D levels are significantly lower in children with latent TB and active TB than in children without TB [52, 71, 72]. Recently, the correlation between low vitamin D and TB disease was confirmed in a multicenter observational study that included 996 children screened for TB [73]. Vitamin D was considered deficient if the serum 25(OH)D level was <25 nmol/L, insufficient between 25 and 50 nmol/L and sufficient at a level >50 nmol/L. Vitamin D levels were significantly lower in children with latent TB than in controls (p = 0.002), in children with active TB than in controls (*p* < 0.0001), and in children with active TB than in those with latent TB (p = 0.001). Moreover, deficient vitamin D levels were found in a higher percentage in the active TB group (n = 18; 40.9 %) compared with the latent TB group (n = 28; 20.3 %) and controls (13.9 %) (*p* < 0.0001), confirming that hypovitaminosis D was significantly associated with TB infection [73]. Finally, a study of 266 Indian children with intrathoracic TB showed that 186 (69.9 %) children were vitamin D deficient (serum 25[OH]D <12 ng/mL), 55 (20.7 %) were insufficient (12 to <20 ng/mL) and 25 (9.4 %) were vitamin D sufficient (≥20 ng/mL) [74]. Levels of 25(OH)D were similar in all three types of intrathoracic TB, and in microbiologically confirmed and probable cases. Levels of 25(OH)D did not significantly affect outcome of the disease. Children who were deficient or insufficient were less likely to convert (i.e., become smear/culture negative) after an intensive phase of antituberculous therapy at two months as compared to those who were 25(OH)D sufficient (*p* < 0.05) [74].

A research on the value of administering vitamin D to children with TB was carried by Marcos et al.; in a randomized study performed in a small number of children (n = 24), vitamin D supplementation (cholecalciferol 1000 IU daily for 8 weeks) was added to TB treatment, leading to better clinical and radiological outcomes compared with the standard treatment alone [75].

Overall, these data showed that low vitamin D is associated with latent TB and active TB. Given the TB burden worldwide and the increase in MDR TB cases, it is very important to find new mechanisms that might reduce the risk of disease and enhance standard treatment efficacy. This highlights the need for further studies to confirm the possible role of vitamin D in the prevention of TB as well as in supportive treatment.

#### Vitamin D and AOM

AOM is a very common problem in pediatric populations, affecting approximately 50 % of infants worldwide in the first year of life. [76, 77]. A subset of children with AOM present with recurrent AOM (rAOM), which is defined as three or more AOM episodes in six months or four or more AOM episodes within 12 months [78]. rAOM is associated with high direct and indirect costs, including antibiotic use and lost working days for parents [78]. Consequently, preventive methods, including prolonged breastfeeding, avoidance of tobacco smoke, nonuse of a pacifier, pneumococcal and influenza vaccination, and supplementation with vitamin D, are extremely important and appear to be effective [79–81].

The first study to suggest an association between vitamin D and AOM was conducted by Sun et al. in rats with rickets [82]. In a subsequent study’s cohort of 475 school-aged children from Bogota, vitamin D deficiency was associated with increased rates of diarrhea with vomiting and earache with fever [83]. More recently, Cayir et al., in a randomized, single-blind, case–control study, concluded that serum calcitriol levels were significantly lower in children diagnosed with AOM than in controls without AOM, suggesting that vitamin D deficiency plays a role in AOM risk [84]. Finally, our group prospectively and blindly randomized 116 children with a history of rAOM to receive oral vitamin D supplementation of 1000 IU/day or placebo for 4 months [85]. The results showed that the number of children with at least one AOM episode during the study period was significantly lower in the treatment group than in the group that received the placebo. Administration of 1000 IU/day of vitamin D restored serum 25(OH)D values of ≥30 ng/mL in most cases and was associated with a significant reduction in the risk of uncomplicated AOM with no benefit for spontaneous otorrhea [85]. Similarly, Cayir et al. demonstrated in a prospective study that serum 25(OH)D levels in patients with rAOM were lower than those in children without rAOM, and a significant reduction in disease frequency was recorded following vitamin D supplementation [86].

In addition, for AOM the overall results suggested that low 25(OH)D serum values are associated with an increased risk of disease. Considering the high frequency of AOM in the pediatric population, studies are needed to identify the serum levels associated with an increased risk of disease and to determine whether vitamin D supplementation can prevent overall AOM cases in children or reduce recurrent episodes in those who already have a history of rAOM.

#### Vitamin D and acute pharyngotonsillitis

Acute pharyngotonsillitis is one of the leading causes of hospital visits during childhood [87, 88]. Most cases are of viral etiology, and among bacterial cases the most important are those caused by *Streptococcus pyogenes* because of their possible complications [89]. Some children have recurrent episodes of pharyngotonsillitis. Recurrent pharyngotonsillitis is defined as at least 7 episodes of pharyngotonsillitis in a year or at least 5 episodes in a year for two consecutive years or at least 3 episodes in a year for three consecutive years [90]. Recurrent pharyngotonsillitis seems to be associated with the bacterial biofilm formation on tonsillar tissue, and antibiotics have low efficacy against them [91]. Preventive therapies are useful in these recurrent cases to avoid tonsillectomy [90].

Vitamin D may have a preventive role in recurrent pharyngotonsillitis by inhibiting the formation of bacterial biofilms, but there are few published studied on this topic. Reid et al. enrolled 33 children in New Zealand who were undergoing tonsillectomy for difficult breathing/sleep apnea and/or recurrent pharyngotonsillitis [92]. They measured 25(OH) vitamin D, iron and zinc levels. Of the 33 patients, 78 % had a 25(OH) vitamin D level <75 nmol/L, and 15.6 % had levels <50 nmol/L. Low 25(OH) vitamin D levels have been linked to risk factors such as darker skin and increased body mass index (BMI) [93]. Yildiz et al. showed that a low serum vitamin D level may be a risk factor for recurrent pharyngotonsillitis because children who had recurrent episodes had serum 25(OH) vitamin levels that were lower than those in healthy children [94]. Since 2012, only one other study has been published on the relationship between serum vitamin D levels and recurrent pharyngotonsillitis, and it was performed in adults. It showed a link between vitamin D deficiency and recurrence of streptococcal pharyngotonsillitis [95].

Although vitamin D supplementation may have a role in the inhibition of biofilm formation, the data are insufficient to allow definitive conclusions to be drawn about the efficacy of this type of supplementation. Further studies would be useful.

#### Vitamin D and rhinosinusitis

Rhinosinusitis (RS) is also extremely common in children, with 0.5–5 % of upper respiratory tract infections progressing to this condition [96]. A few years ago, a retrospective study evaluated serum 25(OH)D levels in children with allergic RS with or without nasal polyposis and found no difference in mean vitamin D3 levels between controls and RS without polyposis, whereas the levels in children with allergic RS with polyposis were lower than the recommended levels [97]. Mullingan et al. confirmed these results in a study performed in adults [98]. In this study, the authors also wanted to determine the effect of cigarette smoke on vitamin D3 levels, conversion, and the regulation of inflammation. All the patients exposed to smoke had lower vitamin D3 levels, and the authors suggested that the reduction of vitamin D3 by cigarette smoke exposure is a novel mechanism through which cigarette smoke induces proinflammatory effects [98].

Because further evidence supports an association of low 25(OH)D with chronic rhinosinusitis in adults [99, 100], and considering that pediatric rhinosinusitis is mainly acute and characterized by the absence of polyposis, studies performed in the pediatric population are urgently needed to clarify the role of vitamin D in children with single or recurrent episodes of acute rhinosinusitis.

#### Vitamin D and acute lower respiratory tract infections: bronchiolitis and pneumonia

Acute lower respiratory tract infection (ALRI) is an important cause of global child mortality, annually accounting for approximately 1.4 million deaths of children younger than 5 years of age [49]. As early as 1975, Salimpour hypothesized a link between vitamin D and pneumonia. Studying 200 rachitic children in Tehran, he found that 43 % also had a history of ALRI [101]. Subsequently, Najada et al. studied a cohort of hospitalized infants with respiratory diseases and found a higher incidence of nutritional rickets [51]. Wayse et al. studied ALRIs in children without rickets admitted to a private hospital in India and recognized a link between sub-clinical vitamin D deficiency, non-exclusive breastfeeding, and increased risk for severe ALRIs [102]. In addition, a meta-analysis of randomized controlled trials showed that prophylactic vitamin D supplementation in pediatric subjects significantly reduced the odds of contracting RTIs (OR, 0.58; 95 % CI, 0.41–0.8) [103]. Interestingly, low cord blood 25(OH)D levels in neonates admitted to neonatal intensive care units have been associated with an increased risk of lower RTIs in the first 2 years of life [104]. To avoid neonatal deficiency and enhance newborns’ respiratory health, it has been proposed that vitamin D supplementation be administered during pregnancy and early childhood [105]. Karatekin et al. found that in 87.5 % of all newborns admitted to neonatal intensive care units, and in 67.5 % of all mothers, serum 25(OH)D concentrations were lower than 20 ng/mL [106]. The 25(OH)D concentrations of newborns were highly correlated with mothers’ serum 25(OH)D concentrations, and for this reason vitamin D supplementation during pregnancy seems beneficial for the neonate.

Bronchiolitis is a viral infectious disease caused mainly by respiratory syncytial virus (RSV) [107]. Some evidence suggests that vitamin D may protect against severe RSV bronchiolitis because in vitro it has been shown that vitamin D decreases the inflammatory response of airway epithelial cells to RSV infection [108]. Moreover, genetic polymorphisms in VDR have been associated with hospitalization for acute bronchiolitis in infancy [109]. A meta-analysis of the existing literature on VDR polymorphisms supported an association between the *Fokl* VDR and RSV severity [110]. In addition, several studies have demonstrated that serum 25(OH)D levels are lower in infants hospitalized for acute bronchiolitis than in with healthy controls [111, 112]. Randolph et al. reported an association between a vitamin D binding protein haplotype and hospitalization for RSV bronchiolitis in infancy in two independent cohorts [113]. By contrast, a Canadian study found no correlation between lower vitamin D levels and risk of hospitalization for ALRI when 25(OH)D concentrations were measured in children hospitalized with bronchiolitis [114].

Some authors hypothesized that vitamin D supplementation in mothers during pregnancy would be useful to prevent acute respiratory infection including bronchiolitis. Belderbos et al. demonstrated that neonates born with a serum 25(OH)D concentration of approximately 50 nmol/L had an important (95 % CI: 1.6-24.9; *p* = 0.01) increase in risk of acute lower RTI due to RSV in the first year of life compared with those who had 25(OH)D concentrations of approximately 75 nmol/L. [115]. In addition, Camargo et al. demonstrated that a higher maternal intake of vitamin D during pregnancy may decrease the risk of recurrent wheeze in early childhood [116]. Overall, these data suggest that vitamin D supplementation for pregnant women and infants may be a useful strategy for preventing and reducing severity of viral respiratory infections that cause bronchiolitis.

Regarding community-acquired pneumonia (CAP), and its morbidity and mortality in children, several interventions have been evaluated to prevent it, including supplementation with vitamin D [117]. Muhe et al. performed a case–control study to determine the role of nutritional rickets in the development of pneumonia, analyzing 521 Ethiopian children with nutritional rickets [118]. Rickets was present in 210 of 500 cases compared with 20 of 500 controls (OR, 22.11; 95 % CI, 11.34–43.12; *p* < 0.0001.). After correction for confounding factors such as family size, birth order, crowding, and months of exclusive breastfeed by logistic regression, the authors concluded that vitamin D or calcium deficiency may be important predisposing factors for pneumonia in children under 5 years of age in developing countries. Later, a case–control study involving 24 Nigerian children showed that not only vitamin D insufficiency but also vitamin D deficiency may have an important role in immune system control [119]. Similar results were found by Haider et al. in 137 Pakistan children [120]. More recently, in 103 children with CAP, Ren et al. found that the mean vitamin D concentration in the group with severe CAP was significantly lower than that in the mild-CAP and control groups (*p* < 0.01), and there was no significant difference between the mild-CAP and control groups (*p* = 0.674) [121]. Zhou et al. measured the serum level of some vitamins and trace elements in a cohort of children with CAP who were randomized into intervention and non-intervention groups with vitamin D supplementation; healthy children of the same age served as controls [122]. Vitamin D serum levels in the CAP group were lower than in children who had CAP and a history of asthma than in the non-asthmatic CAP group or the control group. After vitamin D supplementation, vitamin D serum levels in the asthmatic CAP group increased significantly [122]. VDR polymorphisms seem to be important in relation to CAP risk. A study among children in a Chinese Han population demonstrated that the TT genotype of rs2239185 in the VDR gene may be a genetic risk factor for CAP, and the T allele of rs2239185 may be associated with CAP susceptibility and severity [123].

Studies have been conducted to investigate whether vitamin D supplementation could be useful during the treatment of CAP to improve outcomes. Manaseki-Holland et al. demonstrated no significant difference in the mean number of days to recovery between children with CAP who received a single high dose of vitamin D3 (100,000 IU) and those who received placebo [124, 125]. Nevertheless, the risk of a repeated CAP episode within 90 days was lower in the intervention group than in the placebo group [124]. Choudhary et al. also concluded that short-term oral vitamin D supplementation (1000–2000 IU per day for 5 days) has no beneficial effect on the resolution of severe CAP in children <5 years of age [126]. Finally, a meta-analysis was performed to determine whether vitamin D supplementation has a useful role in the treatment of children <5 years old with acute CAP [127]. The authors concluded that there is no evidence to support therapeutic vitamin D supplementation in the management of children <5 years old with acute CAP. Overall, the available data do not appear conclusive on the role of vitamin D deficiency in increasing CAP risk and do not demonstrate a beneficial effect of vitamin D supplementation on CAP outcomes in the acute disease phase.

### Vitamin D supplementation

Available data support a role for vitamin D deficiency in the risk of pediatric TB, rAOM and severe bronchiolitis, whereas further studies are needed to confirm an association in children with recurrent pharyngotonsillitis, ARS and CAP. Maintenance of adequate vitamin D status could be an effective and inexpensive prophylactic method against these RTIs, but the supplementation regimen has not been clearly defined. In addition to the lack of consensus on whether and in whom there is a need of vitamin D supplementation as well as on the ideal regimen, countries may have different recommendations according to the characteristics of their population. A clarification of the functional effect of VDRs polymorphisms also in relation to ethnicity, sun exposure, skin characteristics, and fat absorption may influence the recommended regimen. At the moment, the supplementation schemes associated with a successful outcome are those which evaluated cholecalciferol 1000 IU daily for 8 weeks in children treated for TB and 1000 IU daily for 4 months in those with a history of rAOM, whereas no benefit was associated with a single high dose of cholecalciferol (100,000 IU) or with a short-term oral supplementation (1000–2000 UI daily for 5 days) in children with CAP. Another approach could be the maternal vitamin D supplementation during pregnancy in order to reduce the future RTIs risk in the offspring, but again the ideal supplementation regimen has not been defined. For this reason, well-defined evidence-based guidelines on the serum 25(OH)D levels associated with disease risk and recommended supplementation regimens are urgently needed.

## Conclusions

Several recent studies have shown that vitamin D has different immunomodulatory properties associated with the risk of RTIs in childhood. In this regard, it is very important to understand the definition of deficiency and insufficiency of vitamin D and when and how to treat this condition. Unfortunately, there is no consensus, although a level of at least 10 ng/mL 25(OH)D is thought to be necessary to promote bone mineralization and calcium homeostasis, and a concentration between 20 ng/mL and 50 ng/mL is considered adequate to provide an immunomodulatory effect [128]. Overall, in children as well as in adults, the term “vitamin D deficiency” indicates values <20 ng/mL, whereas insufficiency is defined as between 20 ng/mL and 30 ng/mL, with at least 30 ng/mL required for optimal health benefits [129–131]. Although hypervitaminosis D is arbitrarily defined as 25(OH)D concentrations >100 ng/mL, symptoms of vitamin D intoxication typically do not manifest until circulating 25(OH)D concentrations rise above 150 ng/mL [132]. With the lack of agreement on the levels of 25(OH)D that constitute sufficiency, there is variability in recommendations for supplementation. Further clinical trials are needed to determine the most appropriate vitamin D supplementation regimen, depending on the type of RTI and taking into account VDR polymorphisms.

## Abbreviations

1,25[OH]D: 1,25-dihydroxyvitamin D3
25(OH)D: 25-hydroxycholecalciferol
ALRI: Acute lower respiratory tract infection
AOM: Acute otitis media
ARS: Acute rhinosinusitis
CAP: Community-acquired pneumonia
CI: Confidence interval
DCs: Dendritic cells
IL: Interleukin
MMPs: Matrix metalloproteinase enzymes
MDR: Multi-drug resistant
OR: Odds ratio
rAOM: Recurrent acute otitis media
RS: Rhinosinusitis
RSV: Respiratory syncytial virus
RTIs: Respiratory tract infections
TB: Tuberculosis
TNF: Tumor necrosis factor
UVB: Ultraviolet B radiation
VDR: Vitamin D receptor

## References

[1] Holick MF (2004). Sunlight and vitamin D for bone health and prevention of autoimmune diseases, cancers, and cardiovascular disease. Am J Clin Nutr.

[2] Norman AW, Bouillon R, Whiting SJ, Vieth R, Lips P (2007). 13th Workshop consensus for vitamin D nutritional guidelines. J Steroid Biochem Mol Biol.

[3] Holick MF, Pang PKT, Schreibman MP (1989). Phylogenetic and evolutionary aspects of vitamin D from phyoplankon to humans. Vertebrate endocrinology: fundamentals and biomedical implications.

[4] Palm TA (1890). The geographical distribution and aetiology of rickets. Practitioner.

[5] Holick MF (2006). Resurrection of vitamin D deficiency and rickets. J Clin Invest.

[6] Rajakumar K, Greenspan SL, Thomas SB, Holick MF (2007). Solar ultraviolet radiation and vitamin D: A historical perspective. Am J Pub Health.

[7] Bischoff-Ferrari HA, Willett WC, Wong JB, Stuck AE, Staehelin HB, Orav EJ (2009). Prevention of nonvertebral fractures with oral vitamin D and dose dependency: a meta-analysis of randomized controlled trials. Arch Intern Med.

[8] Holick MF (2003). Vitamin D: a millennium perspective. J Cell Biochem.

[9] Zittermann A, Gummert JF (2010). Nonclassical vitamin D actions. Nutrients.

[10] Laaksi I, Ruohola JP, Tuohimaa P, Auvinen A, Haataja R, Pihlajamäki H (2007). An association of serum vitamin D concentrations < 40 nmol/L with acute respiratory tract infection in young Finnish men. Am J Clin Nutr.

[11] Ginde AA, Mansbach JM, Camargo CA (2009). Association between serum 25-hydroxyvitamin D level and upper respiratory tract infection in the Third National Health and Nutrition Examination Survey. Arch Intern Med.

[12] Esposito S, Baggi E, Bianchini S, Marchisio P, Principi N (2013). Role of vitamin D in children with respiratory tract infections. Int J Immunopathol Pharmacol.

[13] Marchisio P, Nazzari E, Torretta S, Esposito S, Principi N (2014). Medical prevention of recurrent acute otitis media: an updated overview. Expert Rev Anti Infect Ther.

[14] Norman AW (2008). From vitamin D to hormone D: fundamentals of the vitamin D endocrine system essential for good health. Am J Clin Nutr.

[15] Clemens TL, Adams JS, Henderson SL, Holick MF (1982). Increased skin pigment reduces the capacity of skin to synthesise vitamin D3. Lancet.

[16] Bell NH, Greene A, Epstein S, Oexmann MJ, Shaw W, Shany J (1985). Evidence for alteration of the vitamin D endocrine system in blacks. J Pediatr.

[17] Holick MF (1995). Environmental factors that influence the cutaneous production of vitamin D. Am J Clin Nutr.

[18] Webb AR, Kline L, Holick MF (1988). Influence of season and latitude on the cutaneous synthesis of vitamin D3: exposure to winter sunlight in Boston and Edmonton will not promote vitamin Dı synthesis in human skin. J Clin Endocrinol Metab.

[19] Holick MF (1986). Vitamin D requirements for the elderly. Clin Nutr.

[20] Clemens TL, Zhou X, Myles M, Endres D, Lindsay R (1986). Serum vitamin D2 and vitamin D3 metabolite concentrations and absorption of vitamin D2 in elderly subjects. J Clin Endocrinol Metab.

[21] Stamp TC, Haddad JG, Twigg CA (1977). Comparison of oral 25-hydroxycholecalciferol, vitamin D, and ultraviolet light as determinants of circulating 25-hydroxyvitamin D. Lancet.

[22] Holick MF, Schnoes HK, De Luca HF, Suda T, Cousins RJ (1971). Isolation and identification of 1,25-dihydroxycholecalciferol. A metabolite of vitamin D active in intestine. Biochemistry.

[23] Antoniucci DM, Yamashita T, Portaloe AA (2006). Dietary phosphorus regulates serum fibroblast growth factor-23 concentrations in healthy men. J Clin Endocrinol Metab.

[24] Hewison M, Burke F, Evans KN, Lammas DA (2007). Extra-renal 25-hydroxyvitamin D3-1alphahydroxylase in human health and disease. J Steroid Biochem Mol Biol.

[25] Gray TK, Lester GE, Lorenc RS (1979). Evidence for extra-renal 1α-hydroxylation of 25-hydroxyvitamin D3 in pregnancy. Science.

[26] Barbour GL, Coburn JW, Slatopolsky E, Norman AW, Horst RL (1981). Hypercalcemia in an anephric patient with sarcoidosis: evidence for extrarenal generation of 1,25-dihydroxyvitamin D. N Engl J Med.

[27] Haderslev KV, Jeppesen PB, Sorensen HA, Mortensen PB, Staun M (2003). Vitamin D status and measurements of markers of bone metabolism in patients with small intestinal resection. Gut.

[28] Adorini L, Penna G (2008). Control of autoimmune diseases by the vitamin D endocrine system. Nat Clin Pract Rheumatol.

[29] Thomasset M (1994). Vitamin D and the immune system. Phatol Bio.

[30] Veldman CM, Cantorna MT, De Luca HF (2000). Expression of 1,25 dihydroxyvitamin D(3) receptor in the immune system. Arch Biochem Biophys.

[31] Miyamoto K, Kesterson RA, Yamamoto H, Taketani Y, Nishiwaki E, Tatsumi S (1997). Structural organization of the human vitamin D receptor chromosomal gene and its promoter. Mol Endocrinol.

[32] Jurutka PW, Whitfield GK, Hsieh JC, Thompson PD, Haussler CA, Haussler MR (2002). Molecular nature of the vitamin D receptor and its role in regulation of gene expression. Rev Endocr Metab Disord.

[33] Haussler MR, Whitfield GK, Haussler CA, Hsier JC, Thompson PD, Selznick SH (1998). The nuclear vitamin D receptor: biological and molecular regulatory properties revealed. J Bone Miner Res.

[34] Labuda M, Fujiwara TM, Ross MV, Morgan K, Garcia-Heras J, Ledbetter DH (1992). Two hereditary defects related to vitamin D metabolism map to the same region of human chromosome 12q13–14. J Bone Miner Res.

[35] Uitterlinden AG, Fang Y, Van Meurs JBJ, Pols HAP, JPTM V l (2004). Genetics and biology of vitamin D receptor polymorphism. J Steroid Biochem Mol Biol.

[36] Van Etten E, Mathieu C (2005). Immunoregulation by 1,25-dihydroxyvitamin D3: basic concepts. J Steroid Biochem Mol Biol.

[37] Lemire JM, Archer DC, Beck L, Spielberg HL (1995). Immunosuppressive actions of 1,25-dihydroxyvitamin D3: prefential inhibitions of Th1 functions. J Nutr.

[38] Underwood MA, Bevins CL (2010). Defensin-barbed innate immunity: clinical associations in the pediatric population. Pediatrics.

[39] Di Rosa M, Malaguarnera M, Nicoletti F, Malaguarnera L (2011). Vitamin D3: A helpful immuno-modulator. Immunology.

[40] D’Ambrosio D, Cippitelli M, Cocciolo MG, Mazzeo D, Di Lucia P, Lang R (1998). Inibition of IL-12 production by 1,25-dihydroxyvitamin D3 involvement of NfkB downregulation in transcriptional repression of the p40 gene. J Clin Invest.

[41] Hakim I, Bar-Shavit Z (2003). Modulation of TNF-alpha expression in bone marrow macrophages: involvement of vitamin D response element. J Cell Biochem.

[42] Ryynänen J, Carlberg C (2013). Primary 1,25-dihydroxyvitamin D3 response of the interleukin 8 gene cluster in human monocyte- and macrophage-like cells. Plos One.

[43] Mahon BD, Wittke A, Weaver V, Cantorna MT (2003). The targets of vitamin D depend on the differentiation and activation status of CD4-positive T-cells. J Cell Biochem.

[44] Sun J (2010). Vitamin D and mucosal immune function. Curr Opin Gastroenterol.

[45] Bansal AS, Henriquez F, Sumar N, Patel S (2012). T helper cell subsets in arthritis and the benefits of immunomodulation by 1,25(OH)2 vitamin D. Rheumatol Int.

[46] Al-Daghri NM, Guerini FR, Al-Attas OS, Alokail MS, Alkharfy KM, Draz HM (2014). Vitamin D receptor gene polymorphisms are associated with obesity and inflammosome activity. Plos One.

[47] Shaw NJ, Mughal Z (2013). Vitamin D and child health (extra skeletal aspects). Arch Di Child.

[48] Hewison M (2011). Vitamin D and innate and adaptive immunity. Vitam Horm.

[49] Esposito S, Rigante D, Principi N (2014). Do children’s upper respiratory tract infections benefit from probiotics?. BMC Infect Dis.

[50] Bryce J, Boschi-Pinto C, Shibuya K, Black RE (2005). WHO estimates of the causes of death in children. Lancet.

[51] Najada AS, Habashneh MS, Khader M (2004). The frequency of nutritional rickets among hospitalized infants and its relation to respiratory diseases. J Trop Pediatr.

[52] Williams B, Williams AJ, Anderson ST (2008). Vitamin D deficiency and insufficiency in children with tuberculosis. Pediatr Infect Dis J.

[53] Wilkinson RJ, Llewelyn M, Toossi Z, Patel P, Pasvol G, Lalvani A (2000). Influence of vitamin D deficiency and vitamin D receptor polymorphisms on tuberculosis among Gujarati Asians in west London: a case–control study. Lancet.

[54] Holick MF, Chen TC (2008). Vitamin D deficiency: a worldwide problem with health consequences. Am J Clin Nutr.

[55] Esposito S, Baggi E, Bianchini S, Principi N (2013). Implications of maternal vitamin D deficiency for the fetus, the neonate and the young infant. Eur J Nutr.

[56] Nnoaham KE, Clarke A (2008). Low serum vitamin D levels and tuberculosis: a systematic review and meta-analysis. Int J Epidemiol.

[57] Rios M, Garcıa JM, Sanchez JA, Perez D (2000). A statistical analysis of the seasonality in pulmonary tuberculosis. Eur J Epidemiol.

[58] Schaaf HS, Nel ED, Beyers N, Gie RP, Scott F, Donald P (1996). A decade of experience with *Mycobacterium tuberculosis* culture from children: a seasonal influence on incidence of childhood tuberculosis. Tuber Lung Dis.

[59] Rockett KA, Brookes R, Udalova I, Vidal V, Hill AV, Kwiatkowski D (1998). 1,25-Dihydroxyvitamin D3induces nitric oxide synthase and suppresses growth of *Mycobacterium tuberculosis* in a human macrophage-like cell line. Infect Immun.

[60] Sly LM, Lopez M, Nauseef WM, Reiner NE (2001). 1alpha,25-Dihydroxyvitamin D3-induced monocyte antimycobacterial activity is regulated by phosphatidylinositol 3-kinase and mediated by the NADPH-dependent phagocyte oxidase. J Biol Chem.

[61] Coussens A, Timms PM, Boucher BJ, Venton TR, Ashcroft AT, Skolimowska KH (2009). 1-alpha,25-dihydroxyvitamin D3 inhibits matrix metalloproteinases induced by *Mycobacterium tuberculosis* infection. Immunology.

[62] Liu PT, Stenger S, Li H, Wenzel L, Tan BH, Krutzik SR (2006). Toll-like receptor triggering of a vitamin D-mediated human antimicrobial response. Science.

[63] Martineau AR, Wilkinson KA, Newton SM, Floto RA, Norman AW, Skolimowska K (2007). IFN- {gamma}- and TNF-independent vitamin D-inducible human suppression of Mycobacteria: the role of cathelicidin LL-37. J Immunol.

[64] Yuk JM, Shin DM, Lee HM, Yang CS, Jin HS, Kim KK (2009). Vitamin D3 induces autophagy in human monocytes/macrophages via cathelicidin. Cell Host Microbe.

[65] Selvaraj P, Chandra G, Jawahar MS, Rani MV, Rajeshwari DN, Narayanan PR (2004). Regulatory role of vitamin D receptor gene variants of Bsm I, Apa I, Taq I, and Fok I polymorphisms on macrophage phagocytosis and lymphoproliferative response to mycobacterium tuberculosis antigen in pulmonary tuberculosis. J Clin Immunol.

[66] Roth DE, Soto G, Arenas F, Bautista CT, Ortiz J, Rodriguez R (2004). Association between vitamin D receptor gene polymorphisms and response to treatment of pulmonary tuberculosis. J Infect Dis.

[67] Davies PDO, Nisar M (1990). Racial differences of *Mycobacterium tuberculosis* infection. N Engl J Med.

[68] Davies PDO (1985). A possible link between vitamin D deficiency and impaired host defence to *Mycobacterium tuberculosis*. Tubercle.

[69] Martineau AR, Timms PM, Bothamley GH, Hanifa Y, Islam K, Claxton AP (2011). High-dose vitamin D(3) during intensive-phase antimicrobial treatment of pulmonary tuberculosis: a double-blind randomised controlled trial. Lancet.

[70] Rathored J, Sharma SK, Singh B, Banavaliker JN, Sreenivas V, Srivastava AK (2012). Risk and outcome of multidrug-resistant tuberculosis: vitamin D receptor polymorphisms and serum 25(OH)D. Int J Tuberc Lung Dis.

[71] Gray K, Wood N, Gunasekera H, Sheikh M, Hazelton B, Barzi F (2012). Vitamin D and tubercolosis status in refugee children. Pediatr Infect Dis J.

[72] Gianmaa D, Giovannucci E, Bloom BR, Fawzi W, Burr W, Batbaatar D (2012). Vitamin D, tuberculin skin test conversion, and latent tubercolosis in Mongolian school-age children: a randomized, double-blind, placebo- controlled feasibility trial. Am J Clin Nutr.

[73] Venturini E, Facchini L, Martinez-Alier N, Novelli V, Galli L, de Martino M (2014). Vitamin D and tuberculosis: a multicenter study in children. BMC Infect Dis.

[74] Khandelwal D, Gupta N, Mukherjee A, Lodha R, Singh V, Grewal HM (2014). Vitamin D levels in Indian children with intrathoracic tuberculosis. Indian J Med Res.

[75] Morcos MM, Gabr AA, Samuel S, Kamel M, el Baz M, el Beshry M (1998). Vitamin D administration to tuberculous children and its value. Boll Chim Farm.

[76] Teele D, Klein J, Rosner B (1989). Epidemiology of otitis media during the first seven years of life in children in greater Boston: a prospective, cohort study. J Infect Dis.

[77] Paradise JL, Rockette HE, Colborn DK, Bernard BS, Smith CG, Kurs-Lasky M (1997). Otitis media in 2253 Pittsburgh-area infants: prevalence and risk factors during the first two year of life. Pediatrics.

[78] Greenberg D, Bilenko N, Liss Z (2003). The burden of acute otitis media on the patient and family. Eur J Pediatr.

[79] Marchisio P, Nazzari E, Torretta S, Esposito S, Principi N (2014). Medical prevention of recurrent acute otitis media: an updated overview. Expert Rev Anti Infect Ther.

[80] Pappas DE, Owen Hendley J (2003). Otitis media. A scholarly review of the evidence. Minerva Pediatr.

[81] Gravel JS, Karma P, Casselbrant ML, Marchisio P, Andalibi A, Passali D (2005). Recent advances in otitis media. Diagnosis and screening. Ann Otol Rhinol Laryngol Suppl.

[82] Sun H, Tao Z, Li X, Fang J (1997). The spontaneous otitis media in rickety rats. Hunan Yi Ke Da Xue Xue Bao.

[83] Thornton KA, Marin C, Mora-Plazas M, Villamor E (2013). Vitamin D deficiency associated with increased incidence of gastrointestinal and ear infections in school-age children. Pediatr Infect Dis J.

[84] Cayir A, Turan MI, Okzan O, Cayir Y (2014). Vitamin D levels in children diagnosed with acute otitis media. J Park Med Assoc.

[85] Marchisio P, Consonni D, Baggi E, Zampiero A, Bianchini S, Terranova L (2013). Vitamin D supplementation reduces the risk of acute otitis media in otitis-prone children. Pediatr Infect Dis J.

[86] Cayir A, Turan MI, Ozkan O, Cayir Y, Kaya A, Davutoglu S (2014). Serum vitamin D levels in children with recurrent otitis media. Eur Arch Otorhinolaryngol.

[87] Alan L, Bisno MD (1996). Acute pharyngitis: etiology and diagnosis. Pediatrics.

[88] Armstrong GL, Pinner RW (1999). Outpatient visits for infectious diseases in the United States, 1980 through 1996. Arch Intern Med.

[89] Pelucchi C, Grigoryan L, Galeone C, Esposito S, Huovinen P, ESCMID Sore Throat Guideline Group (2012). Guideline for the management of acute sore throat. Clin Microbiol Infect.

[90] Paradise JL, Bluestone CD, Colborn DK, Bernard BS, Rockette HE, Kurs-Lasky M (2002). Tonsillectomy and adenotonsillectomy for recurrent throat infection in moderately affected children. Pediatrics.

[91] Nazzari E, Torretta S, Pignataro L, Marchisio P, Esposito S (2015). Role of biofilm in children with recurrent upper respiratory tract infections. Eur J Clin Microbiol Infect Dis.

[92] Reid D, Morton R, Salkeld L, Bartley J (2011). Vitamin D and tonsil disease--preliminary observations. Int J Pediatr Otorhinolaryngol.

[93] Aydın S, Aslan I, Yıldız I, Ağaçhan B, Toptaş B, Toprak S (2011). Vitamin D levels in children with recurrent tonsillitis. J Pediatr Otorhinolaryngol.

[94] Yildiz I, Unuvar E, Zeybek U, Toptas B, Cacina C, Toprak S (2012). The role of vitamin D in children with recurrent tonsillopharyngitis. Ital J Pediatr.

[95] Nseir W, Mograbi J, Abu-Rahmeh Z, Mahamid M, Abu-Elheja O, Shalata A (2012). The association between vitamin D levels and recurrent group A streptococcal tonsillopharyngitis in adults. Int J Infect Dis.

[96] Esposito S, Marchisio P, Tenconi R, Tagliaferri L, Albertario G, Patria MF (2012). Diagnosis of acute rhinosinusitis. Pediatr Allergy Immunol.

[97] Mulligan JK, White DR, Wang EW, Sansoni SR, Moses H, Yawn RJ (2012). Vitamin D3 deficiency increases sinus mucosa dendritic cells in pediatric chronic rhinosinusitis with nasal polyps. Otolaryngol Head Neck Surg.

[98] Mulligan JK, Nagel W, O’Connell BP, Wentzel J, Atkinson C, Schlosser RJ (2014). Cigarette smoke exposure is associated with vitamin D3 deficiencies in patients with chronic rhinosinusitis. J Allergy Clin Immunol.

[99] Wang LF, Lee CH, Chien CY, Chen JY, Chiang FY, Tai CF (2013). Serum 25-hydroxyvitamin D levels are lower in chronic rhinosinusitis with nasal polyposis and are correlated with disease severity in Taiwanese patients. Am J Rhinol Allergy.

[100] Schlosser RJ, Soler ZM, Schmedes GW, Storck K, Mulligan JK (2014). Impact of vitamin D deficiency upon clinical presentation in nasal polyposis. Int Forum Allergy Rhinol.

[101] Salimpour R (1975). Rickets in Tehran. Arch Dis Child.

[102] Wayse V, Yousafzai A, Mogale K, Filteau S (2004). Association of subclinical vitamin D deficiency with severe acute lower respiratory infection in Indian children under 5 y. Eur J Clin Nutr.

[103] Charan J, Goyal JP, Saxena D, Yadav PJ (2012). Vitamin D for prevention of respiratory tract infections: a systematic review and meta-analysis. Pharmacol Pharmacother.

[104] Mohamed WA, Al-Shehri MA (2013). Cord blood 25-hydroxyvitamin D levels and the risk of acute lower respiratory tract infection in early childhood. J Trop Pediatr.

[105] Dinlen N, Zenciroglu A, Beken S, Dursun A, Dilli D, Okumus N. Association of vitamin D deficiency with acute lower respiratory tract infections in newborns. J Matern Fetal Neonatal Med. 2015, Epub March 19. 10.3109/14767058.2015.1023710 25786473

[106] Karatekin G, Kaya A, Salihoğlu O, Balci H, Nuhoğlu A (2009). Association of subclinical vitamin D deficiency in newborns with acute lower respiratory infection and their mothers. Eur J Clin Nutr.

[107] Bosis S, Esposito S, Niesters HG, Crovari P, Osterhaus AD, Principi N (2005). Impact of human metapneumovirus in childhood: comparison with respiratory syncytial virus and influenza viruses. J Med Virol.

[108] Hansdottir S, Monick MM, Lovan N, Powers L, Gerke A, Hunninghake GW (2010). Vitamin D decreases respiratory syncytial virus induction of NF-kappaB-linked chemokines and cytokines in airway epithelium while maintaining the antiviral state. J Immunol.

[109] Roth DE, Jones AB, Prosser C, Robinson JL, Vohra S (2008). Vitamin D receptor polymorphisms and the risk of acute lower respiratory tract infection in early childhood. J Infect Dis.

[110] McNally JD, Sampson M, Matheson LA, Hutton B, Little J (2014). Vitamin D receptor (VDR) polymorphisms and severe RSV bronchiolitis: a systematic review and meta-analysis. Pediatr Pulmonol.

[111] McNally JD, Leis K, Matheson LA, Karuananyake C, Sankaran K, Rosenberg AM (2009). Vitamin D deficiency in young children with severe acute lower respiratory infection. Pediatr Pulmonol.

[112] Roth DE, Shah R, Black RE, Baqui AH (2010). Vitamin D status and acute lower respiratory infection in early childhood in Sylhet, Bangladesh. Acta Paediatr.

[113] Randolph AG, Yip WK, Falkenstein-Hagander K, Weiss ST, Janssen R, Keisling S (2014). Vitamin D-binding protein haplotype is associated with hospitalization for RSV bronchiolitis. Clin Exp Allergy.

[114] Roth DE, Jones AB, Prosser C, Robinson JL, Vohra S (2009). Vitamin D status is not associated with the risk of hospitalization for acute bronchioitis in early childhood. Eur J Clin Nutr.

[115] Belderbos ME, Houben ML, Wilbrink B, Lentjes E, Bloemen EM, Kimpen JL (2011). Cord blood vitamin D deficiency is associated with respiratory syncytial virus bronchiolitis. Pediatrics.

[116] Camargo CA, Ingham T, Wickens K, Thadhani R, Silvers KM, Epton MJ (2011). Cord-blood 25-hydroxyvitamin D levels and risk of respiratory infection, wheezing, and asthma. Pediatrics.

[117] Esposito S, Principi N (2012). Unsolved problems in the approach to pediatric community-acquired pneumonia. Curr Opin Infect Dis.

[118] Muhe L, Lulseged S, Mason KE, Simoes EA (1997). Case–control study of the role of nutritional rickets in the risk of developing pneumonia in Ethiopian children. Lancet.

[119] Oduwole AO, Renner JK, Disu E, Ibitoye E, Emokpae E (2010). Relationship between vitamin D levels and outcome of pneumonia in children. West Afr J Med.

[120] Haider N, Nagi AG, Khan KM (2010). Frequency of nutritional rickets in children admitted with severe pneumonia. J Pak Med Assoc.

[121] Ren J, Sun B, Miao P, Feng X (2013). Correlation between serum vitamin D level and severity of community acquired pneumonia in young children. Zhongguo Dang Dai Er Ke Za Zhi.

[122] Zhou W, Zuo X, Li J, Yu Z. Effects of nutrition intervention on the nutritional status and outcomes of paediatric patients with pneumonia. Minerva Pediatr. 2015, Epub Mar 31. 25823620

[123] Li W, Guo L, Li H, Sun C, Cui X, Song G (2015). Polymorphism rs2239185 in vitamin D receptor gene is associated with severe community-acquired pneumonia of children in Chinese Han population: a case–control study. Eur J Pediatr.

[124] Manaseki-Holland S, Qader G, Isaq Masher M, Bruce J, Zulf Mughal M, Chandramohan D (2010). Effects of vitamin D supplementation to children diagnosed with pneumonia in Kabul: a randomised controlled trial. Trop Med Int Health.

[125] Manaseki-Holland S, Maroof Z, Bruce J, Mughal MZ, Masher MI, Bhutta ZA (2012). Effect on the incidence of pneumonia of vitamin D supplementation by quarterly bolus dose to infants in Kabul: a randomised controlled superiority trial. Lancet.

[126] Choudhary N, Gupta P (2011). Vitamin D supplementation for severe pneumonia: a randomized controlled trial. Indian Pediatr.

[127] Das RR, Singh M, Panigrahi I, Naik SS (2013). Vitamin d supplementation for the treatment of acute childhood pneumonia: a systematic review. ISRN Pediatr.

[128] Cranney A, Horsley T, O’Donnell S, Weiler H, Puil L, Ooi D (2007). Effectiveness and safety of vitamin D in relation to bone health. Evid Rep Technol Assess (Full Rep).

[129] Ross AC, Taylor CL, Yaktine AL, Del Valle HB (2001). Committee to review dietary reference intakes for vitamin D and calcium. Institute of Medicine: Dietary Reference Intakes for Calcium and Vitamin D.

[130] Urashima M, Segawa T, Okazaki M, Kurihara M, Wada Y, Ida H (2010). Randomized trial of vitamin D supplementation to prevent seasonal influenza A in schoolchildren. Am J Clin Nutr.

[131] Inamo Y, Hasegawa M, Saito K, Hayashi R, Ishikawa T, Yoshino Y (2011). Serum vitamin D concentrations and associated severity of acute lower respiratory tract infections in Japanese hospitalized children. Pediatr Int.

[132] Holick MF, Binkley NC, Bishoff-Ferrari HA, Gordon CM, Hanley DA, Heaney RP (2011). Evaluation, treatment, and prevention of vitamin D deficiency: an Endocrine Society clinical practice guideline. J Clin Endocrinol Metab.

